# A Systematic Review on the Efficacy of Bisphosphonates on Osteogenesis Imperfecta

**DOI:** 10.7759/cureus.86549

**Published:** 2025-06-22

**Authors:** Rohan R Datir, Rohit R Datir, Pooja R Datir, Nasser Heyrani

**Affiliations:** 1 Medicine, California University of Science and Medicine, Colton, USA; 2 Biology, New Jersey Institute of Technology, Newark, USA; 3 Orthopedic Surgery, Oak Tree Orthopedics, Corona, USA

**Keywords:** bisphosphonate use, bone mineral density, fracture rate, fracture risk, oi osteogenesis imperfecta

## Abstract

Osteogenesis imperfecta (OI) is a rare genetic disorder that causes frequent fractures. Bisphosphonates play a key role in managing OI. This manuscript examines the comparative effectiveness of alendronate, neridronate, olpadronate, pamidronate, risedronate, and zoledronic acid on fracture rate reduction, increases in lumbar spine (LS) bone mineral density (BMD), and adverse effects compared to placebos and each other. A PubMed search using specific keywords for bisphosphonates and OI yielded 21 sources, from which data about fracture rates, fracture risk, and/or LS BMD were collected. The inclusion criteria consisted of randomized controlled trials involving bisphosphonates to treat OI with data regarding fracture rates, fracture risk, and/or BMD, and the exclusion criteria were any sources that did not meet such standards. A one-way analysis of variance (ANOVA) was conducted to assess the significance of differences among bisphosphonates. Neridronate, olpadronate, and risedronate had a lower fracture risk and fracture rate than their placebo counterparts. Olpadronate demonstrated a markedly lower fracture rate compared to its placebo, and neridronate similarly showed a substantially reduced fracture risk relative to its placebo. Risedronate was effective but less so than the other two. Pamidronate showed the largest overall increase in LS BMD, while alendronate demonstrated the highest placebo-adjusted ratio. Despite ANOVA testing finding insignificant differences between drugs, except for fracture risk, limited data constrained the analysis. Adverse effects varied: alendronate caused the most gastrointestinal distress, zoledronic acid and neridronate caused illness-like symptoms, risedronate had illness-like and gastrointestinal symptoms, and pamidronate was linked to severe effects, including death. This analysis highlighted the efficacy and safety profiles of bisphosphonates in the treatment of OI. Neridronate and olpadronate were highly effective in reducing fracture risk and rates, and olpadronate demonstrated superior efficacy in reducing fracture rates. Future research should focus on large, diverse samples, detailed fracture and BMD data, and comparisons across multiple bisphosphonates to refine treatment strategies.

## Introduction and background

Osteogenesis imperfecta (OI) is a rare genetic disorder, primarily characterized by increased bone fragility and susceptibility to fracture [1]. It arises from mutations in genes responsible for the production and structure of type I collagen, such as Collagen Type I Alpha 1 (COL1A1) and Collagen Type I Alpha 2 (COL1A2), which form the foundation of the bone matrix [2]. This defect reduces bone density, ligamentous laxity, and impairs mechanical properties, leading to frequent fractures, skeletal deformities, short stature, and bone pain [3]. Depending on the genetic mutation and severity, OI is classified into different types, ranging from mild (Type I) to perinatal lethal forms (Type II) and progressively deforming types (Type III and IV) [4].

The management of OI is multidisciplinary, encompassing surgical intervention, physiotherapy, and pharmacotherapy [1]. Bisphosphonates, the pharmacological cornerstone, have gained attention for their ability to inhibit bone resorption and improve bone strength [5]. These drugs, widely used in other metabolic bone diseases such as osteoporosis and Paget’s disease, have been repurposed for OI treatment due to their profound impact on bone mineral density (BMD) and reduction in fracture risk [5]. Understanding the mechanism of action and clinical applications of this disorder provides valuable insights into optimizing treatment [5].

Mechanism of action of bisphosphonates

Bisphosphonates are synthetic analogs of pyrophosphate, a naturally occurring molecule that regulates bone metabolism [6]. Their structure contains two phosphate groups bound to a central carbon atom, forming a P-C-P backbone [5]. This configuration allows bisphosphonates to bind strongly to hydroxyapatite crystals in bone, localizing their activity to areas of active bone remodeling [5]. Their mechanism of action can be divided into two primary pathways: inhibition of osteoclast activity and reduction of bone turnover [5].

Inhibition of Osteoclast Activity

Bisphosphonates exert their effects by disrupting the function of osteoclasts, the cells responsible for bone resorption [7]. They are absorbed by osteoclasts during bone resorption and interfere with intracellular processes, leading to cytoskeletal disruption. The nitrogen-containing bisphosphonates impair the osteoclast’s ability to adhere to the bone surface by inhibiting farnesyl pyrophosphate synthase (FPPS), a key enzyme in the mevalonate pathway [8]. This disruption prevents the formation of a ruffled border, which is essential for bone resorption [9]. Additionally, bisphosphonates induce programmed cell death in osteoclasts, reducing their population and activity [10]. It is important to acknowledge that although osteoclast activity is suppressed, osteoblastic activity is not necessarily increased. However, suppression of osteoclastic activity provides benefits to patients with OI, as the net effect is a reduction in BMD decrease.

Reduction in Bone Turnover

Bisphosphonates inhibit osteoclast-mediated bone resorption, which can lead to a shift in the bone remodeling process. This shift may favor bone formation over resorption, which can combat imbalances between these two processes, as found in conditions such as OI [5]. This preference enhances BMD and strengthens both cortical and trabecular bone, thereby reducing fracture rates [11]. Moreover, bisphosphonates bind to hydroxyapatite, preventing mineral loss and reducing bone brittleness [12].

Numerous bisphosphonates - notably, alendronate, neridronate, olpadronate, pamidronate, risedronate, and zoledronic acid - have demonstrated a reduction in fracture rates and/or an increase in BMD in patients with OI [13]. Despite bisphosphonates being standard therapy, their long-term efficacy, safety, and comparative advantages warrant further exploration. Given the recent increase in literature, there is a need to comprehensively review the breadth of bisphosphonate pharmacotherapy to determine their intra-class comparative efficacy and their efficacy versus placebo. This manuscript examines the reduction of fracture rates, increases in lumbar spine (LS) BMD, decreases in the mean fracture rate per subject, and adverse effects of numerous bisphosphonates commonly used in the treatment of OI. Fracture rate is the mean number of fractures per person, fracture risk is the percentage of participants who experienced a fracture during the study, and LS BMD is a measure of the mineral content found in the lumbar spine of a patient.

## Review

Methods

A systematic literature search was conducted using PubMed to identify studies evaluating the efficacy of bisphosphonates in treating OI. The search string used was: (“bisphosphonate” OR “alendronate” OR “clodronate” OR “etidronate” OR “ibandronate” OR “olpadronate” OR “risedronate” OR “pamidronate” OR “tiludronate” OR “zoledronate”) AND (“osteogenesis imperfecta” OR “brittle bone disease”). The search was conducted in January 2025 and included studies from the database's inception through December 2024. No language, age, or sex filters were applied. The initial search yielded 578 results.

To narrow the scope to high-quality evidence, results were filtered for randomized controlled trials (RCTs) using PubMed’s built-in filters, which reduced the total to 23 articles. Articles were then screened for relevance by reviewing titles, abstracts, and full texts. Inclusion criteria were human studies involving patients diagnosed with OI, use of bisphosphonates as the primary intervention, and reported quantitative outcomes related to fracture rate, fracture risk, and/or BMD. Of the 23 RCTs, two were excluded due to irrelevance, resulting in 21 included studies. Although some studies reported BMD measurements at multiple skeletal sites (e.g., femur, total body), the vast majority provided LS BMD. Given this inconsistency across studies and the need for comparable outcome metrics, we chose to focus exclusively on LS BMD as the most commonly reported and clinically meaningful measure. We acknowledge that this decision may limit generalizability to other skeletal regions, and this limitation is discussed further in the discussion section.

**Figure 1:**
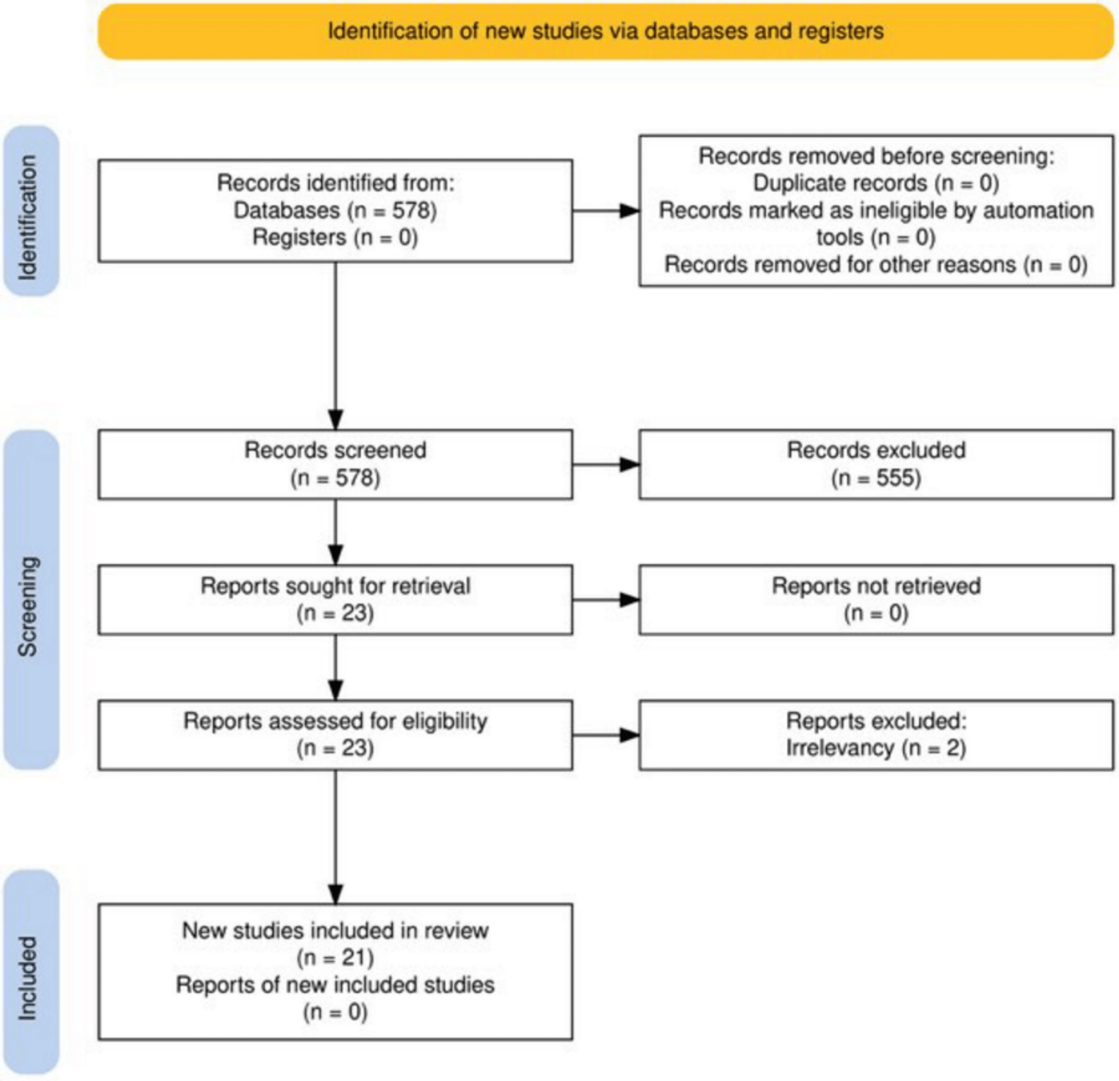
The PRISMA flow diagram, which depicts the identification, appraisal, and screening process

A flow diagram (Figure 1), consistent with PRISMA guidelines [14], illustrates the study selection process.

For studies reporting LS BMD or fracture rates, data were extracted as group-level means and standard deviations. When multiple bisphosphonate types were compared, results were grouped by drug class (e.g., pamidronate, alendronate, zoledronate). A one-way analysis of variance (ANOVA) was performed to compare average LS BMD changes, fracture rates and reductions, and fracture risks and reductions across these treatment groups.

Because the analysis utilized study-level summary data rather than individual patient-level data, this was a between-group comparison of aggregated outcomes. Additionally, weighting by sample size was not performed for the ANOVA test. While we did not formally test ANOVA assumptions (such as normality or homogeneity of variances), we selected ANOVA based on the continuous nature of the dependent variables and the clear independence of the study groups. We acknowledge that applying ANOVA to heterogeneous study-level data has limitations, including the assumption of equal variances and unweighted group comparisons, which are discussed later.

Missing data were handled by excluding studies that did not report a specific outcome (e.g., LS BMD or fracture rate) from the corresponding analysis. No imputation was performed. ANOVA results were interpreted as exploratory and hypothesis-generating, not as confirmatory statistical tests.

Results

Data collation from the 21 included studies [15-35] was done on Microsoft Excel (Microsoft Corp., Redmond, WA) and the parameters noted are given in Table 1.

**Table 1:** Data collected from the 21 reviewed sources LS BMD: Lumbar spine bone mineral density; N/A: Not applicable; rGH: recombinant growth hormone; IV: Intravenous; GI: Gastrointestinal

Author	Drug name	Dose	Combination agents	Comparator drug	Method of delivery	Sample size	Fracture risk	Fracture rate	LS BMD	Adverse effects
Sakkers et al. (2004) [15]	Olpadronate	10 mg/m^3^ daily	500 mg/m^3^ calcium, 400 IU cholecalciferol	Placebo	Oral	34 (16 drug, 18 placebo)	50% for olpadronate, 78% for placebo	1.125 for olpadronate, 2.78 for placebo	N/A	GI distress
Kok et al. (2007) [16]	Olpadronate	10 mg/m^2^ daily for 2 years	500 mg/m^2^ daily calcium, 400 IU daily cholecalciferol	Placebo	Oral	34 (16 drug, 18 placebo)	60% for olpadronate, 71% for placebo	1.2 for olpadronate, 4.33 for placebo	N/A	N/A
Ward et al. (2011) [17]	Alendronate	5 mg daily for children <40 kg, 10 mg daily for children ≥40 kg, for 2 years	Calcium, vitamin D	Placebo	Oral	139 (109 drug, 30 control)	N/A	2.136 for alendronate, 2.091 for placebo	50.7% increase for alendronate, 11.9% increase for placebo	Pain, GI distress, extraskeletal ossification, leukopenia, agitation, and syringomyelia/platybasia
Lv et al. (2018) [18]	Alendronate, zoledronic acid	70 mg/year for alendronate, 5 mg/year for zoledronic acid	500 mg calcium, 200 mg IU vitamin D3	N/A	Oral for alendronate, IV for zoledronic acid	136 (90 for alendronate, 46 for zoledronic acid)	35.5% for aledronate, 25.9% for zoledronic acid	N/A	60.01% increase for alendronate, 62.04% increase for zoledronic acid	GI distress, feer, flu-like symptoms, and chills for aledronate. Fever, myalgia, flu-like symptoms, and chills for zoledronic acid
Dimeglio et al. (2005) [19]	Pamidronate, alendronate	1 mg/kg per day for 3 days/4 months for pamidronate, 1 mg/kg/day for alendronate, for 8-12 months	N/A	N/A	IV for pamidronate, oral for alendronate	12 (6 for pamidronate, 6 for alendronate)	N/A	N/A	29.2% increase for pamidronate, 44.5% increase for alendronate	N/A
Senthilnathan et al. (2008) [20]	Pamidronate, pamidronate	12 mg/kg/year of pamidronate, 6 mg/kg/year of pamidronate	N/A	N/A	IV	12 (6 for 12 mg/kg/year, 6 for 6 mg/kg/year)	50% for 12 mg/kg/year, 66% for 6 mg/kg/year	2.33 for 12 mg/kg/year, 4.5 for 6 mg/kg/year	108% increase for 5 mg/kg/year, 153% increase for 12 mg/kg/year	Death
Antoniazzi et al. (2010) [21]	Neridronate	2 mg/kg/3 months	800-1000 ng/day calcium, vitamin D levels maintained above 20 mg/mL	Neridronate + rGH	IV	30 (15 for neridronate, 15 for neridronate + rGH)	N/A	0.6 in neridronate, 0.66 in neridronate + rGH	N/A	N/A
Seikaly et al. (2005) [22]	Alendronate	5 mg/day for subjects <30 kg, 10 mg/day for subjects ≥30 kg for 12 months, alternating with placebo	1000-1300 mg/day calcium, 800-1200 mg/day phosphorus, and 400 IU/day vitamin D	Placebo	Oral	17 (some received 12 months placebo, followed by 12 months alendronate, others received in opposite order)	N/A	1.214 during placebo, 0.286 during alendronate	N/A	GI distress
DiMeglio et al. (2006( [23]	Alendronate, pamidronate	1 mg/kg/day for alendronate, 3 mg/kg/4 months for pamidronate	N/A	N/A	Oral for alendronate, IV for pamidronate	18 (9 for alendroante, 9 for pamidronate)	N/A	3.4 for alendronate, 4.2 for pamidronate	N/A	Fever, myagia, GI distress for pamidronate
Xu et al. (2016) [24]	Alendronate, zoledronic acid	70 mg/week for aledronate, 5 mg/year for zoledronic acid for 24 months	500 mg calcium, 200 IU vitamin D3	N/A	Oral for alendronate, IV for zoledronic acid	52 (33 for alendronate, 19 for zoledronic acid)	33% for alendronate, 15.79% for zoledronic acid	0.512 for alendronate, 0.263 for zoledronic acid	10.5% increase for alendronate, 11.3% increase for zoledronic acid	GI distress, nausea for alendronate, fever and body aches for zoledronic acid
Bishop et al. (2013) [25]	Risedronate	2.5 mg/day for subjects 10-30 kg, 5 mg/day for subjects >30 kg	500-1000 mg calcium, 200-600 IU vitamin D, appropriate for weight	Placebo for one year, followed by risedronate for two years	Oral	143 (94 for risedronate, 49 for placebo + risedronate)	53% for risedronate, 65% for placebo + risedronate	1.38 for risedronate, 2.34 for placebo + risedronate	N/A	Pain, headache, arthralgia, nausea, pyrexia, flu-like symptoms
Chevrel et al. (2006) [26]	Alendronate	10 mg/day	1000 mg calcium, 800 IU vitamin D3 daily	Placebo	Oral	64 (31 for alendronate, 33 for placebo)	38.71% for alendronate, 29.03% for placebo	1.7 for alendroante, 1.727 for placebo	10.1% increase for alendronate, 0.7% increase for placebo	GI distress
Antoniazzi et al. (2006) [27]	Neridronate	2 mg/kg	400 UI/day vitamin D, 600 mg/day calcium minimum	Placebo	IV	20 (5 treated immediately (group A), 5 treated after 6 months (group B), 10 for placebo (group C))	N/A	4.4 for group A, 8.4 for group B, 12.5 for group C	N/A	Fever
Barros et al. (2012) [28]	Pamidronate, zoledronic acid	1 mg/kg/day for pamidronate, 0/025-0.05 kg/day for zoledronic acid for 2 days per 3-4 months	Calcium and vitamin D	N/A	IV	23 (11 for pamidronate, 12 for zoledronic acid)	N/A	3.24 for pamidronate, 3.73 zoledronic acid	9.22% increase for pamidronate, 6.52% increase for zoledronic acid	Fever, GI distress, nausea, myalgia, skin rash for pamidronate, fever, nausea, myalgia, headache for zoledronic acid
Ward et al. (2005) [29]	Alendronate	35 mg for subjects <40 kg, 70 mg for subjects >40 kg as tablets, on top of IV 125 ug for all subjects	Calcium	N/A	IV and oral	12 below 40 kg, 12 above 40 kg	N/A	N/A	N/A	Headache, nausea, fever, and pain
Letocha et al. (2005) [30]	Pamidronate, rGH	10 mg/m^2^/day for 3 days every 3 months for pamidronate, 0.06 mg/kg/day for 6 days/week for rGH	500 mg/day calcium for subjects <10 years old, 1000 mg/day calcium for subjects >10 years old	Placebo	IV for pamidroante and rGH	18 (9 for pamidronate, 9 for placebo, 4 in each group had rGH injections)	66.67% for pamidronate, 44.44% for placebo	1.67 for pamidronate, 0.78 for placebo (baseline was also higher for pamidronate)	N/A	N/A
Adami et al. (2003) [31]	Neridronate	100 mg/250 mL saline for 30 mins/ 3 months	50,000 IU vitamin D/month if levels 20 ng/mL, calcium levels maintained >1000 mg/day	Placebo	IV	46 (31 for neridronate, 15 for placebo)	N/A	0.032 for neridronate, 0.133 for placebo	3% increase for neridronate, 0% increase for placebo	Flu-like symptoms
Gatti et al. (2005) [32]	Neridronate	100 mg/250 mL saline/3 months	50,000 IU/month vitamin D2 if levels <20 ng/mL3	Placebo	IV	26 (13 for neridronate, 13 for placebo)	N/A	N/A	N/A	N/A
Rauch et al. (2009) [33]	Risedronate	15 mg/week for subjects <40 kg, 30 mg/week for subjects >40 kg	Calcium or vitamin D supplementation provided if daily intake was <800 mg or <400 IU, respectively	Placebo	Oral	26 (13 for risedronate, 13 for placebo)	53.85% for risedronate, 46.15% for placebo	0.846 for risedronate, 0.846 for placebo	N/A	N/A
Gatti et al. (2004) [34]	Neridronate	2 mg/kg/3 months	600-800 and 1000 mg calcium according to age, 50,000 IU/month vitamin D2 if vitamin D levels <20 ng/mL	Placebo	IV	64 (42 for neridronate, 22 for placebo)	27% for neridronate, 45% for placebo	0.309 for neridronate, 0.818 for placebo group	30% increase for neridronate, 3.5-5.7% increase for placebo	N/A
Muratore et al. (2013) [35]	Neridronate, alendronate, risedronate	25 mg/month for neridronate, 70 mg/week for alendronate, 35 mg/week for risedronate for 12 months	800 IU oral vitamin D3 and 1 g calcium daily, 10-15 mg/week of methotrexate, non-steroidal anti-inflammatory drug and proton pump inhibitor supplemented per week, along with 5 mg methylprednisolone at unspecified frequency for 12 months	N/A	Intramuscular for neridronate, oral for alendroante and risedronate	87 (30 for neridronate, 27 for alendronate, 30 for risedronate)	N/A	N/A	3.12% increase for neridronate, 3.01% increase for alendronate, 2.92% increase for risedronate	GI distress for alendronate and risedronate, flu-like symptoms and fever for neridronate

Moreover, all 21 reviewed sources and all other sources referenced in this manuscript were cited using Zotero (Corporation for Digital Scholarship, Vienna, VA, US). Out of the included studies (Table 1), nine (42.86%) involved alendronate, six (28.57%) involved neridronate, two (9.52%) involved olpadronate, five (23.81%) involved pamidronate, three (14.29%) involved risedronate, and three (14.29%) involved zoledronic acid.

Fracture risk is the likelihood that a subject would experience a fracture, calculated by dividing the number of subjects who experienced a fracture by the total number of subjects. For papers that compared a bisphosphonate to a placebo, the reduction in fracture risk was the difference between the placebo’s and the bisphosphonate’s fracture risk. Fracture rate, on the other hand, is the average number of fractures a subject experienced, calculated by dividing the number of fractures recorded among subjects by the number of subjects. Similar to fracture risk, the reduction in fracture rate was the difference between the placebo’s and the bisphosphonate’s fracture rate. 

In this analysis, fracture risk, decrease in fracture risk, fracture rate, and decrease in fracture rate were weighted by sample size. For example, if study A had five subjects with a fracture risk of 2% and study B had 10 subjects with a fracture risk of 8%, the weighted fracture risk would be 6% due to the following calculation: ((5 subjects × 0.02 risk) + (10 subjects × 0.08 risk)) ÷ (5 subjects + 10 subjects) = 6%. The purpose of weighting is to ensure that the average fracture risk and fracture rate for all studies combined could be calculated to accurately reflect each drug’s ability to reduce fracture risk and fracture rate.

Fracture Risk Reduction Efficacy

**Figure 2:**
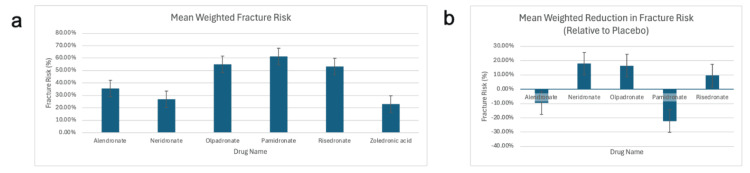
Mean fracture risk, weighted by sample size (a) Mean fracture risk for alendronate, neridronate, olpadronate, pamidronate, risedronate, and zoledronic acid, weighted by sample size; (b) Mean fracture risk for placebo minus mean fracture risk weighted by sample size for alendronate, neridronate, olpadronate, pamidronate, and risedronate [15-35].

Figure 2a presents the mean weighted fracture risk for each drug and Figure 2b shows the mean weighted difference in fracture risk between each drug and placebo.

Although olpadronate and risedronate were associated with relatively high absolute fracture risks, both demonstrated a reduction in fracture risk compared to their respective placebo groups. Neridronate also had a lower fracture risk compared to placebo, and its fracture risk in absolute terms was not as high as olpadronate and risedronate. Thus, neridronate was associated with a low fracture risk and demonstrated the most favorable reduction in fracture risk relative to placebo among all the bisphosphonates. Conversely, pamidronate had the highest fracture risk and showed an increase in fracture risk compared to placebo, making it the least effective agent in this regard.

While these observations might suggest some relationship between a drug’s absolute fracture risk and its relative benefit, the pattern is inconsistent. For instance, olpadronate showed a high fracture risk but still demonstrated benefit over placebo. Therefore, we conclude that no consistent correlation exists between a drug’s absolute fracture risk and its reduction in fracture risk relative to placebo.

Zoledronic acid was excluded from Figure 2b due to the absence of placebo-controlled studies in the dataset (no study comparing zoledronic acid to placebo met the inclusion criteria).

Fracture Rate Reduction

**Figure 3:**
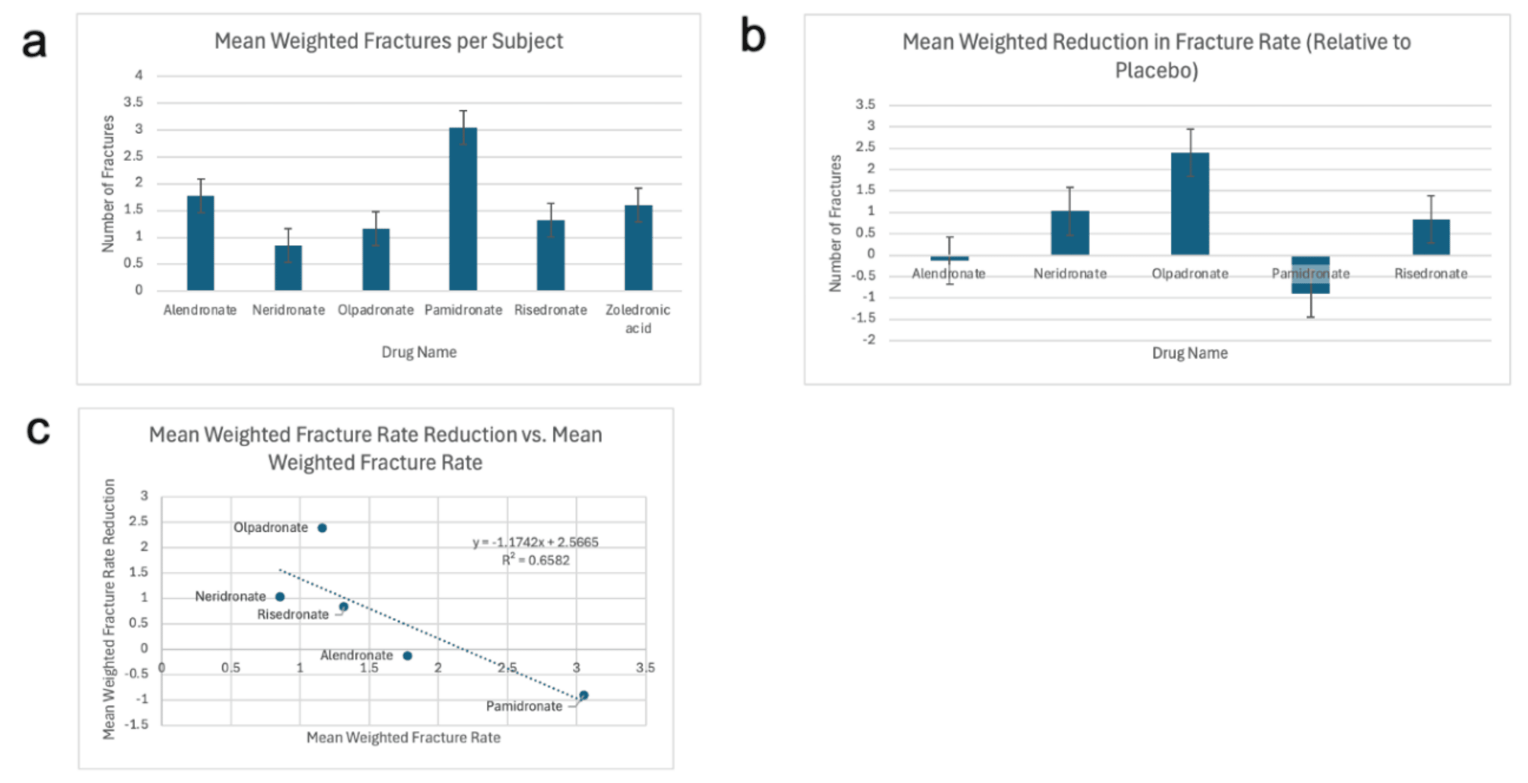
Mean fracture rate for each drug, weighted by sample size (a) Mean fracture rate for alendronate, neridronate, olpadronate, pamidronate, risedronate and zoledronic acid, weighted by sample size; (b) Mean fracture rate for placebo minus mean fracture rate weighted by sample size for alendronate, neridronate, olpadronate, pamidronate, and risedronate; (c) Scatterplot of the relationship between mean weighted fracture rate reduction and mean weighted fracture rate [15-35].

Figures 3a-3c show the mean weighted fracture rate for each drug, the fracture rate difference between placebo and each drug, and a scatterplot exploring the relationship between absolute fracture rate and reduction relative to placebo, respectively.

A general inverse trend was observed. Drugs with higher fracture rates tended to show smaller reductions in fracture rate relative to placebo. Neridronate was an exception; despite having the lowest fracture rate, its relative reduction in fracture rate compared to placebo was smaller than that of olpadronate.

Overall, neridronate and olpadronate were the most favorable drugs based on both low fracture rates and reductions compared to placebo. Zoledronic acid was excluded from Figures 3b, 3c due to the absence of placebo-controlled data (no relevant studies).

LS BMD Increase

**Figure 4:**
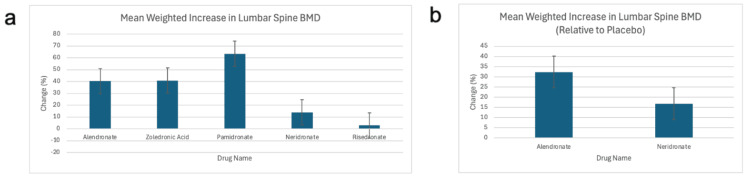
Mean increase in LS BMD for each drug and difference versus placebo, weighted by sample size LS BMD: Lumbar spine bone mineral density; (a) Mean increase in LS BMD for alendronate, zoledronic acid, pamidronate, neridronate, and risedronate, weighted by sample size; (b) Mean increase in LS BMD for placebo minus mean increase in LS BMD for alendronate and neridronate, weighted by sample size [15-35].

Figures 4a, 4b present the mean LS BMD increase for each drug, weighted by sample size, and the LS BMD difference between each drug and placebo, respectively.

All drugs showed an increase in LS BMD, with pamidronate demonstrating the largest increase, followed by alendronate and zoledronic acid. Neridronate and risedronate showed the smallest increases.

However, only studies of alendronate and neridronate included placebo groups for the LS BMD comparison, so the LS BMD increase relative to placebo could not be evaluated for other drugs. This limits the interpretation of comparative efficacy across all treatments.

It is also important to acknowledge the apparent disconnect between the LS BMD increase and clinical outcomes such as fracture risk and rate. For example, pamidronate had the highest LS BMD gain but also had the worst performance in terms of fracture risk. This discrepancy underscores the complexity of translating bone density improvements into clinical fracture prevention and suggests that LS BMD alone may not be a reliable surrogate endpoint for fracture outcomes in OI.

ANOVA Testing

The results of one-way ANOVA testing are summarized in Table 2.

**Table 2:** ANOVA tests for fracture risk, fracture risk reduction, fracture rate, fracture rate reduction, and LS BMD increase ANOVA: Analysis of variance; LS BMD: Lumbar spine bone mineral density [15-35]

Test	P-value	Test statistic	Effect size	Null hypothesis
Risk	0.00155933	14.887858	Large (2.92)	Rejected
Risk reduction	0.313949	1.778331	Large (0.94)	Not rejected
Rate	0.744431	0.53833	Large (0.42)	Not rejected
Rate reduction	0.337785	1.334696	Large (0.76)	Not rejected
LS BMD increase	0.254206	1.561395	Large (0.65)	Not rejected

ANOVA was conducted to compare the means of five outcome variables: fracture risk, fracture risk reduction, fracture rate, fracture rate reduction, and LS BMD increase. Data were grouped by bisphosphonate type, and sample size weighting was not applied in this analysis.

Drugs with only a single data point for a given variable were excluded from that specific ANOVA. The following exclusions were made:

1. Fracture risk ANOVA: All six drugs included (no exclusions).

2. Fracture risk reduction ANOVA: Zoledronic acid excluded (no placebo-controlled studies).

3. Fracture rate ANOVA: All six drugs included.

4. Fracture rate reduction ANOVA: Zoledronic acid excluded.

5. LS BMD increase ANOVA: Zoledronic acid excluded.

The null hypothesis was rejected only for fracture risk (p<0.05), indicating a statistically significant difference in fracture risk across drugs. For all other outcomes, the ANOVA yielded non-significant p-values.

Despite some notably large mean differences in outcomes between drugs (e.g., pamidronate vs. neridronate in LS BMD increase), these were not statistically significant. We caution that large effect sizes in the absence of statistical significance should be interpreted carefully, especially given the limited number of studies per drug and the exclusion of zoledronic acid from numerous ANOVA tests. These limitations suggest that the ANOVA findings are exploratory in nature and should not be interpreted as definitive comparative efficacy outcomes. However, they provide numerous insights into the efficacy of bisphosphonate therapy in OI.

Adverse Effects

Alendronate was strongly associated with gastrointestinal distress and illness-like symptoms and thus had the greatest number of adverse effects. However, previous research on the side effects of alendronate suggests a possible link between alendronate and gastrointestinal distress, but such symptoms can be controlled with limited dosing [36]. Gastrointestinal distress, such as mild upper gastrointestinal effects, was present after alendronate administration [25]. Literature regarding aches/pain after alendronate is conflicting; some studies indicate that alendronate does not exacerbate pain, while others report otherwise [16,25]. There is limited research about leukopenia after alendronate, with only one source mentioning it [36]. 

All the adverse effects with zoledronic acid and neridronate were typically illness-like symptoms. Flu-like symptoms, such as fever, were reported for both zoledronic acid and neridronate (Table 1). Other literature also demonstrates flu-like symptoms and myalgia for zoledronic acid [37], and fever after neridronate administration [38].

Pamidronate had a diverse range of symptoms. Flu-like symptoms after pamidronate are common, such as fever [39]. Moreover, one subject from Senthilnathan et al. [19] died while on pamidronate. In contrast, previous literature suggests that pamidronate does not increase mortality in patients with OI [40]. 

Risedronate had a combination of illness-like symptoms and gastrointestinal symptoms, although other studies indicate that risedronate is free of serious adverse events [41]. Although there was one instance of GI distress for olpadronate, there is limited literature regarding olpadronate's adverse effects in patients with OI.

Table 3 demonstrates the cumulative adverse effects of each drug.

**Table 3:** Total adverse effects by drug [15-35]

Adverse effect	Alendronate	Neridronate	Olpadronate	Pamidronate	Risedronate	Zoledronic acid	Total
Aches/Pain	2	0	0	0	1	1	4
Agitation	1	0	0	0	0	0	1
Arthralgia	0	0	0	0	1	0	1
Chills	1	0	0	0	0	1	2
Death	0	0	0	1	0	0	1
Extraskeletal ossification	1	0	0	0	0	0	1
Fever	2	1	0	2	1	3	9
Flu-like symptoms	1	2	0	0	1	2	6
Gastrointestinal distress	6	0	1	2	2	0	11
Headache	1	0	0	0	1	1	3
Leukopenia	1	0	0	0	0	0	1
Myalgia	0	0	0	2	0	2	4
Nausea	2	0	0	1	1	1	5
Skin rash	0	0	0	1	0	0	1
Syringomyelia/Platybasia	1	0	0	0	0	0	1
Total	19	3	1	9	8	11	51

Table 4 depicts the risk of bias using Joanna Briggs Institute (JBI)'s critical appraisal checklists [42] for each study.

**Table 4:** Risk of bias assessment using Joanna Briggs Institute (JBI)'s appraisal checklists RCT: Randomized controlled trial; ITT: Intention to treat; OI: Osteogenesis imperfecta

Author (Year)	Study type	Overall risk of bias	Justification summary
Sakkers et al. (2004) [15]	RCT	Low	Double-blind RCT with complete reporting and central allocation.
Kok et al. (2007) [16]	RCT	Low	Secondary analysis from Sakkers et al.; maintains same low bias profile.
Ward et al. (2011) [17]	RCT	Low	Well-conducted double-blind RCT with ITT analysis and assessor blinding.
Lv et al. (2018) [18]	RCT	Moderate	Randomized but open-label; no ITT or allocation concealment.
DiMeglio et al. (2005) [19]	RCT	Moderate	Open-label trial; no allocation concealment or ITT.
Senthilnathan et al. (2008) [20]	RCT	Moderate	Open-label with unclear assessor blinding and no ITT.
Antoniazzi et al. (2010) [21]	RCT	Moderate	Unblinded, no allocation concealment, no ITT.
Seikaly et al. (2005) [22]	RCT	Moderate	Open-label design, no ITT, unclear assessor blinding.
DiMeglio et al. (2006) [23]	RCT	Low	Randomized trial with blinded assessors and complete follow-up.
Xu et al. (2016) [24]	Observational study	High	Not a randomized trial; lacks blinding, randomization, and ITT.
Bishop et al. (2013) [25]	RCT	Low	High-quality double-blind RCT with ITT and proper allocation.
Chevrel et al. (2006) [26]	RCT	Low	Double-blind RCT with robust outcome reporting and statistical methods.
Antoniazzi et al. (2006) [27]	RCT	Moderate	Randomized but open-label and no ITT analysis.
Barros et al. (2012) [28]	RCT	Moderate	Open-label RCT with good follow-up but no blinding or ITT.
Ward et al. (2005) [29]	RCT	Low	Double-blind pharmacokinetics trial with full follow-up.
Letocha et al. (2005) [30]	RCT	Moderate	Randomized with blinded assessors, but no group blinding or ITT.
Adami et al. (2003) [31]	RCT	Moderate	Open-label trial with reliable measurements but no ITT or allocation concealment.
Gatti et al. (2005) [32]	RCT	Moderate	Randomized design, but lacked blinding and ITT.
Rauch et al. (2009) [33]	RCT	Low	Well-designed double-blind RCT with proper statistical analysis.
Gatti et al. (2005) [34]	RCT	Moderate	Open-label pediatric RCT with good follow-up but no ITT.
Muratore et al. (2013) [35]	RCT	Moderate	Adherence-focused RCT without blinding or ITT; population not OI.

Discussion

For both fracture risk reduction and fracture rate reduction, neridronate, olpadronate, and risedronate consistently demonstrated favorable outcomes, while alendronate and pamidronate were associated with comparatively less favorable or even negative effects on fracture outcomes [15-35]. Specifically, olpadronate and neridronate performed similarly in fracture risk reduction, but olpadronate was more effective in reducing fracture rate. Risedronate showed modest effectiveness for both measures.

In contrast, pamidronate yielded the greatest increase in LS BMD, and alendronate demonstrated the largest LS BMD improvement relative to placebo, though this was based on a small number of studies. Given the mixed profile of these drugs, poor fracture outcomes but strong LS BMD gains, they may still be viable options for patients with high fracture susceptibility at the lumbar spine, particularly when the therapeutic goal is short-term bone density enhancement. However, the clinical relevance of BMD improvements without parallel reductions in fracture rates is uncertain, and this potential disconnect should be carefully weighed during treatment selection.

Although ANOVA testing did not reveal statistically significant differences between the drugs for most outcomes (except fracture risk), this likely reflects the limited statistical power due to small sample sizes and the exclusion of drugs with only a single data point per analysis. Therefore, the ANOVA results should be interpreted as exploratory, rather than a definitive evidence of treatment equivalence or superiority.

Regarding adverse effects, alendronate was associated with the highest reported gastrointestinal distress. Existing literature suggests that these symptoms are largely preventable by adhering to dosing instructions, making patient education crucial, especially for individuals with pre-existing gastrointestinal conditions [15-36]. Zoledronic acid and neridronate were associated with flu-like symptoms, while risedronate caused both gastrointestinal and illness-like side effects, potentially complicating recovery in patients already experiencing systemic illness. Pamidronate was linked to various symptoms, including one pneumonia-related death; however, the causal relationship between the drug and the death was not definitively clarified in the paper. 

Future Applications and Limitations

Although this review explores multiple aspects of bisphosphonate therapy and bone health, several limitations must be acknowledged. First, this paper does not examine the potential differences in bisphosphonate efficacy across demographic covariates such as age, sex, race/ethnicity, comorbidities, or combination therapies. Future studies should consider stratified RCTs or large observational cohort studies that compare bisphosphonate effectiveness across demographic groups, ideally reporting fracture incidence per 100 patient-years, BMD changes over time, and treatment adherence or adverse event profiles by subgroup.

Additionally, no included studies investigated the combined or sequential use of multiple bisphosphonates, which could offer insights into long-term efficacy, rebound effects, or drug interactions. Future trials might explore head-to-head or crossover designs to evaluate the outcomes of switching or combining bisphosphonates in patients with OI.

A number of entries in Table 1 were marked as 'N/A,' indicating missing outcome data such as fracture risk, fracture rate, or LS BMD. This restricted our ability to conduct comprehensive comparisons or include all drugs in the ANOVA testing. Moreover, the limited number of eligible RCTs, many with small sample sizes ranging from 12 to 40 participants, reduced the statistical power and generalizability of the findings. Notably, no single study included all the bisphosphonates reviewed in this paper, and each drug’s results were drawn from separate studies with differing methodologies, follow-up durations, and patient populations. This introduces a risk of between-study heterogeneity and limits the reliability of drug-to-drug comparisons. Furthermore, several included studies may contain baseline imbalances or uncontrolled confounding.

## Conclusions

This analysis highlights the varied efficacy and safety profiles of bisphosphonates in the treatment of OI. Neridronate and olpadronate generally showed favorable outcomes in reducing both fracture risk and fracture rate, and olpadronate appeared particularly effective in fracture rate reduction. However, it is important to note that the number of studies evaluating these drugs was limited, and the data were often incomplete, making these findings preliminary and in need of further validation. Risedronate also demonstrated effectiveness, but to a lesser extent. For LS BMD improvements, pamidronate showed the greatest absolute increase, and alendronate had the highest placebo-adjusted increase, though this was based on a small number of studies. Given the limited availability of placebo-controlled BMD data, conclusions about comparative BMD efficacy should be interpreted with caution, as they may not be generalizable across broader patient populations.

Adverse effects remain an important factor in treatment decisions. Alendronate was associated with the highest number of gastrointestinal side effects, and neridronate, zoledronic acid, and risedronate were linked to illness-like symptoms. Pamidronate was associated with several adverse outcomes, including one case of mortality; however, the study did not confirm a causal relationship between pamidronate and the death, and this should be interpreted carefully. Overall, while this review supports the potential role of bisphosphonates in improving clinical outcomes in OI, the variability in study design, limited sample sizes, and missing data reduce the strength of these conclusions.
